# Study on the outdoor thermal comfort of college students under different activity intensities in a high-altitude climate zone

**DOI:** 10.3389/fpubh.2024.1365470

**Published:** 2024-03-18

**Authors:** Yingzi Zhang, Xiaobo Zhang, Jiaqin Han, Xinxing Liu

**Affiliations:** School of Architecture, Southwest Jiaotong University, Chengdu, China

**Keywords:** high altitude, college students, outdoor thermal comfort, activity intensity, cold climate

## Abstract

**Introduction:**

Research on the outdoor thermal comfort (OTC) of a university campus is beneficial to the physical and mental health of college students.

**Methods:**

In this study, the OTC of students attending Tibet University in Lhasa, which experiences high-altitude cold climate conditions, under different activity intensities was studied using field measurements and a questionnaire survey.

**Results:**

With the increase in activity intensity, the comfort physiologically equivalent temperature (PET) value gradually increased in summer, while the comfortable PET value gradually decreased in winter. The most comfortable PET value is 17.6°C in summer and 11.5°C in winter. The neutral PET of Tibetan college students during outdoor activities in summer was 16.3°C, and the neutral PET of outdoor activities in winter was 12.1°C. Gender and ethnicity had different effects on thermal sensation under different activity intensities. Under vigorous-intensity activities, PET in winter and summer had the greatest influence on thermal sensation. The situation was different under moderate-intensity activity. PET had the greatest influence on thermal sensation in summer, and Tmrt had the greatest influence on thermal sensation in winter.

**Discussion:**

These findings provide a basis for an improved design of the outdoor environment under different outdoor activity intensities in high-altitude areas.

## 1 Introduction

College students are facing a health crisis in their growth stage. As of 2014, China’s sub-health population reached 70% (1). Based on the statistics of different educational backgrounds, the number of sub-healthy college students, i.e., with poor health, accounted for 8% of the total number of respondents (2). Studies have shown that the Body Mass Index (BMI) of young people in China is significantly higher than that of older adults, which represents a higher risk of obesity (3). Moreover, over the past two decades, the physical activity of Chinese young people has declined by 32% (4). The data show that the level of physical activity of college students decreases significantly in winter, especially in high-altitude and high-latitude areas (5). Compared with mild climate areas, it is more difficult to carry out physical activity on university campuses in high-altitude cold areas and there are health risks for college students due to a lack of physical activity.

The current outdoor thermal comfort research sites are mainly urban streets, squares, parks, waterfronts, and settlements. The climate zones mainly include Mediterranean climate (6, 7), desert climate (8), subtropical climate (9), tropical climate (10), and continental climate (11, 12). Of the few studies on campus thermal comfort, most were conducted in temperate, subtropical, hot summer and cold winter, and extremely cold climates. Among these studies, in the indoor area of the campus, Liu et al. (13) investigated the thermal comfort of students in a classroom of the university with natural ventilation under temperate climate conditions; they found that the neutral temperature was 20.6°C and the comfortable temperature was 19.5–21.8°C. For the outdoor area of the campus, Huang et al. (14) compared the thermal comfort of the semi-open and outdoor open areas of the University of Hong Kong campus in a subtropical climate. The results showed that the neutral PET was 21.0°C in the semi-outdoor area and 22.7°C in the outdoor open area. Zhao et al. (15) found that the neutral SET index was 23.9°C in the subtropical Guangzhou outdoor area, which was similar to the results of Xi et al. (16). They studied the outdoor thermal comfort (OTC) of the campus outdoor space in the same climate zone. China has a vast territory. However, there are few studies on outdoor thermal comfort in campuses similar to extremely cold cities in Tibet, and there is a lack of quantitative research on the influence of outdoor thermal environment factors on human thermal comfort. Therefore, this paper takes this as an opportunity to conduct qualitative and quantitative research on the key issue of outdoor thermal comfort in university campuses in high altitude areas.

Studies have shown that a lack of exercise or sedentary behavior increases the risk of physical and physiological diseases, such as depression and cancer (17–19). Furthermore, physical activities can promote metabolism and change the thermal comfort of the human body, so different activity intensities result in different thermal comfort sensations. Different activity intensities have different thermal comfort requirements, and the influence of the microclimate on thermal comfort is also different under different intensity activities. Furthermore, subjects have different thermal expectations of the environment when they are at different activity intensities, resulting in differences in the acceptable temperature range of the human body. Zheng (20), from Chongqing University, used a combination of experimental research and a questionnaire survey, as well as the methods of mathematical statistics and physiological analysis, to study the influence of the activity level on human thermal sensation in a neutral and cool temperature environment. The higher the ambient temperature, the smaller the effect of the activity level on human thermal sensation. Studies in parks in cold cities have shown that the stronger the activity intensity, the stronger the demand for temperature and humidity in the activity area. The sensitivity of dynamic activities to microclimate is different from that of static activities, and perceptions of the microclimate elements of dynamic activities are also different from those of static activities (21). In addition, due to individual differences, even in the same thermal environment, subjects of different genders and ethnic groups may have different thermal perceptions. Studies have found that women are more sensitive to the thermal environment than men (6, 22). However, the results are inconsistent. In addition to personal factors, ethnic differences can also affect current feelings of heat. Another focus of this study is to analyze the impact of thermal comfort from a gender and ethnic perspective. Therefore, this study improves the related research on activity intensity and different genders for human thermal comfort, which can provide guidance for the subsequent improvement of human outdoor activity comfort.

In this study, we investigated the thermal response of college students participating in different intensity (light, moderate, and intense) sports activities on a campus in Lhasa, a high-altitude cold region in China. The main objectives of our research are to determine the following:

1. Effects of individual factors on the thermal response to different intensities of activity;
2. Thermal benchmark of college students under different activity intensity levels;
3. Effect of meteorological factors on the thermal response under different intensities of activity.

## 2 Methodology

### 2.1 Study area

**Figure 1:**
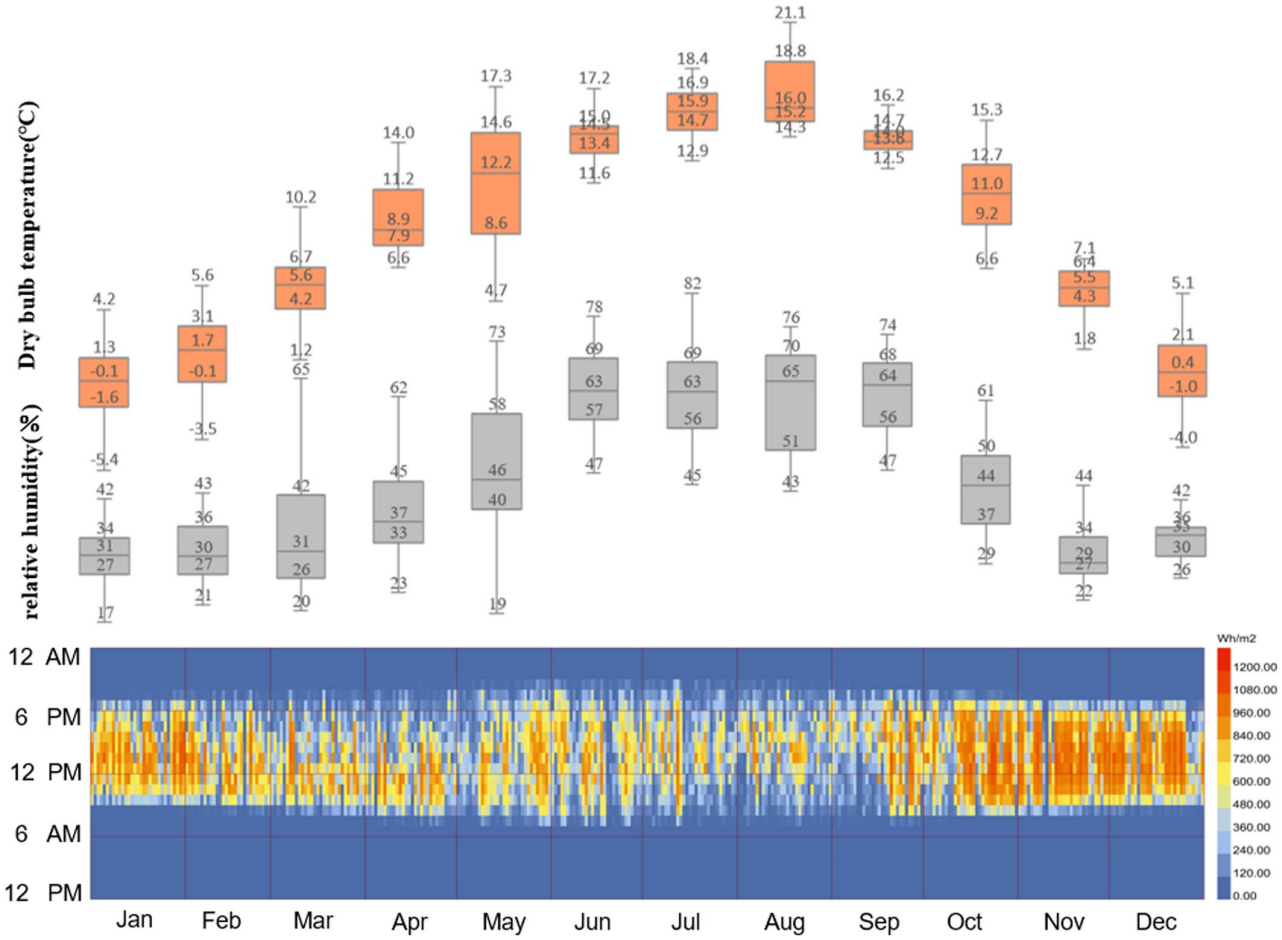
Solar radiation intensity, dry bulb temperature, and relative humidity data for Lhasa.

Lhasa is the largest city on the Qinghai-Tibet Plateau, with an average altitude of 3,650 m. As shown in Figure 1, the typical climate characteristics are low pressure, high solar radiation (SR), low average temperature, low relative humidity, and high wind speed (23). According to the Köppen climate zone, Lhasa has a warm summer and a dry, cold winter (Dwb) (24) The SR intensity in Lhasa is strong, and the annual sunshine duration (SD) can reach more than 3,000 h.

### 2.2 Experimental design

The research idea of this thesis is as follows, in the first step, through the field research method, several typical spaces with different types of intensity activities were selected in Najin Campus of Tibet University. In the second step, the subjective thermal responses of the subjects under different intensity activities and the current level of thermal environment parameters were obtained through field measurements combined with questionnaires in the typical spaces. In the third step, Spearman correlation analysis (Spearman correlation analysis) and multiple regression analysis [Binary Logistic (Bin)] were used to explore the mechanisms of association between PET and thermal sensation, environmental factors and subjective thermal response under different personal factors, as well as the thermal benchmark under different intensity activity levels was investigated. Finally, we propose space optimization design strategies for outdoor spaces with different activity intensities.

#### 2.2.1 Selection of field experimental test points

**Figure 2:**
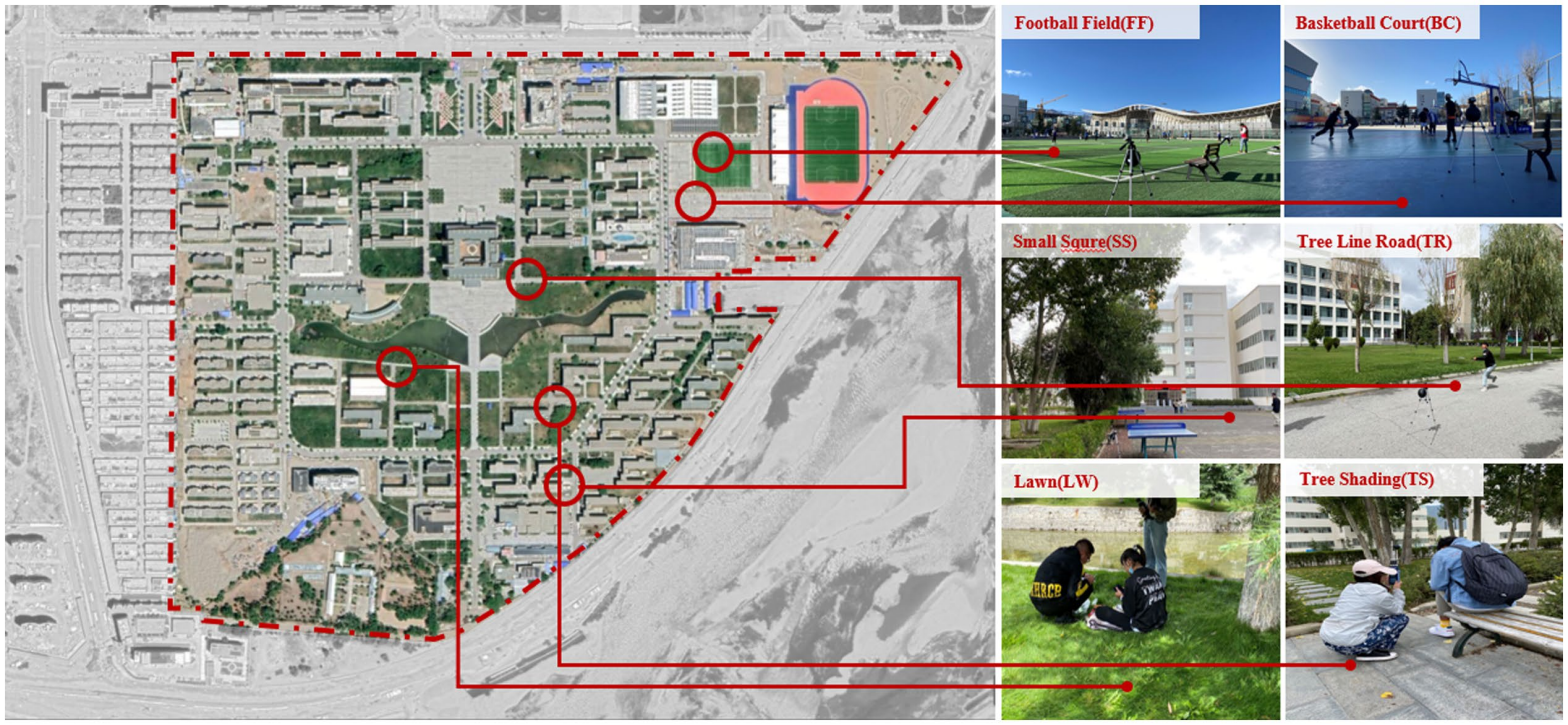
Distribution map of measuring points.

The field experiment was conducted at the Najin Campus of Tibet University in Lhasa. The types of student activities in Tibet University can be divided into different intensity categories. The principle of reliability and representativeness of test data, the football field, basketball field, the small square in front of a dormitory building, shaded road, lawn and shaded space of Tibet University were selected for field experiments with different activity intensities. The measuring points are shown in Figure 2.

#### 2.2.2 Experimental procedures

According to the Compendium of Physical Activities (25) coding table, sitting and standing are light-intensity exercise; table tennis and badminton are moderate-intensity sports; and football and basketball are heavy-intensity sports. The experiment was divided into two parts: high-intensity activity test and moderate-intensity activity test on the first day, and low-intensity activity test on the second day. The average value of light intensity activity was 1.65 met, the average value of moderate intensity activity was 4.75 met, and the average value of vigorous intensity activity was 8.5 met. At the beginning of the experiment, the students waited for 5 min and then moved according to the corresponding intensity at the corresponding place. The students included both men and women who were either Tibetan or Han. Studies have shown that thermal sensation tends to be stable within 15–20 min after a change in metabolic rate (26). Therefore, in this study, participants were allowed to rest for 15 min after the completion of the corresponding activity intensity, and then subjective thermal response data and meteorological data were collected (Table 1).

**Table 1:** Instrument specification and accuracy.

Instrument	Image	Precision	Experimental height	Recording mode	Black globe diameter
Solar radiation recorder (SPN1)	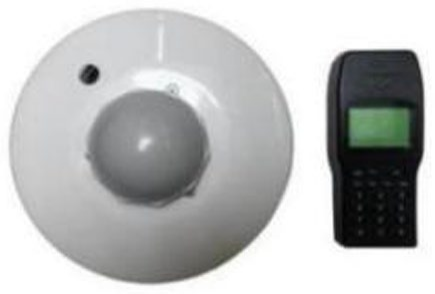	±5% ±10 W.m^2^	1,100 mm	15 min interval	80 mm
Portable microclimate information meter	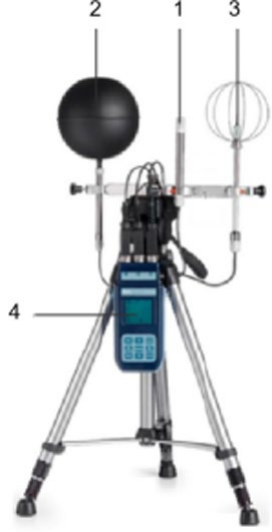	Air temperature: 0.01°C Air humidity: 0.01% Black globe temperature: 0.01°C 3. Air velocity (AP32032): 0.01 m/s	1,100 mm	15 min interval	80 mm

In addition, due to the uncontrollable factor that the activity location was outdoors, there were Tibetan college students in the activity location in addition to the tested students. In this regard, the experimenters also collected a large number of test data of college students who carried out corresponding activities in different intensity activity sites. Ethical approval for this study was also provided by the Ethics Committee of the Southwest Jiaotong University, School of Architecture.

#### 2.2.3 Questionnaire survey

The questionnaire mainly included three parts, as shown in Table 2. The first part was the basic characteristics of the college students, including gender, height, weight, ethnicity, attire, and exercise status (27). The second part was the subjective voting of college students (28), including thermal sensation voting (TSV), thermal comfort voting (TCV), and thermal acceptability voting (TAV). The third part consisted of the microclimate data at the completion of the test, including air temperature (T_a_), relative humidity (RH), SR, globe temperature (T_g_), and air velocity (V_a_).

**Table 2:** Questionnaire used in this study.

Part 1	Biological sex	□ Male □ Female
	Height	() cm
	Weight	() kg
	Nationality	□ Tibetan □ Han
	Wearing condition	Upper body: □ T-shirt □ Sweater □ Thin coat □ Padded jackets □ Down jackets
		Lower body: □ Long trousers □ Short trousers
		Feet: □ Sports shoes □ Others
	Current activity status	□ Light intensity □ Moderate intensity □ Vigorous intensity
Part 2	Thermal sensation	□ Cold (−3) □ Cool (−2) □ Slightly cool (−1) □ Neutral (0) □ Slightly warm (1) □ Warm (2) □ Hot (3)
	Thermal comfort	□ Very comfortable (0) □ Quite comfortable (1) □ Slightly comfortable (2) □ Slightly uncomfortable (3) □ Uncomfortable (4)
	Thermal acceptability	□ Very unacceptable (−2) □ Just unacceptable (−1) □ Just acceptable (0) □ Acceptable (1) □ Very acceptable (2)
	Thermal preference	Temperature: □ Lower (−1) □ No change (0) □ Higher (1) Wind speed: □ Lower (−1) □ No change (0) □ Higher (1) Humidity: □ Lower (−1) □ No change (0) □ Higher (1) Solar radiation: □ Lower (−1) □ No change (0) □ Higher (1)
Part 3	Climate data measurements	Temperature () Black ball temperature () Wind speed () Relative humidity () Solar radiation ()

### 2.3 Pet calculation

In this study, physiologically equivalent temperature (PET) was used to evaluate the OTC. Mean radiation temperature (MRT) is an important part of the calculation of PET (29). The calculation formula of Tmrt is as follows:


$$T_{mrt}={\left[{\left(T_{g}+273.15\right)}^{4}+\frac{1.1\times 10^{8}V_{a}^{0.6}}{\varepsilon D^{0.4}}\times \left(T_{g}-T_{a}\right)\right]}^{1/4}-273.15$$

where D is the diameter of a black globe (mm); ε is the radiation emissivity of the black globe; T_g_ is the globe temperature (°C); T_a_ is air temperature (°C); and V_a_ is air velocity (m/s).

PET is calculated using Rayman software. The software can perform corrections at low air pressure and high-altitude conditions. During computation, parameters such as air temperature, relative humidity, air speed, mean radiant temperature, region, longitude, latitude, and altitude are required as input (24). PET can be calculated using RayMan software (RayMan 1.2 (2000), Meteorological Institute of the University of Freiburg, Germany), which has been used for outdoor climate calculations (30). In this study, the MRT of each measuring point was calculated by the globe temperature measurement method, and then, T_a_ (°C), RH (%), V_a_ (M/s), T_mrt_ (w/m^2^), gender, clothing thermal resistance, and activity intensity were input to Rayman to calculate PET.

### 2.4 Analytical way

#### 2.4.1 Spearman correlation analysis

Spearman correlation analysis is a nonparametric statistical method used to assess the strength and direction of the relationship between two variables (31). It is typically used to measure the monotonic relationship between two variables without regard to the distribution of data between the variables or whether the variables are proportional, and the magnitude of the correlation coefficient determines the strength of the relationship between the variables.

#### 2.4.2 Regression analysis

In order to measure the thermal benchmarks at different intensity activity levels, the method of Binary Logistic (Bin) was used in this project, which is a statistical analysis method that transforms the relationship between the independent variable and the dependent variable into a probability value, thus predicting and interpreting the dependent variable (32). This method can better demonstrate the mechanism of the influence of different factors on thermal sensation.

## 3 Research results

### 3.1 Descriptive statistics

#### 3.1.1 Basic information of subjects

The questionnaire survey took into account gender, ethnicity, intensity of activity and other objective factors, and a total of 643 valid questionnaires were returned, including 319 in summer and 324 in winter. The subjects were students of Tibet University, and the subjects were healthy and free from diseases, while all of them had more than three months’ living experience in Tibet University to ensure the subjects’ adaptation to the Tibetan environment. Among them, 54% were Tibetan students and 46% were Han Chinese students, and the average weight of males was 74 kg and that of females was 54 kg. The average height of males was 173 cm and that of females was 162 cm. A total of 700 questionnaires were distributed in these study, 656 questionnaires were collected, and 643 questionnaires were valid, with an effective rate of 91.9%.

To facilitate the calculation, the thermal resistance calculation formula recommended by the 2017 ASHRAE standards (33) was used to calculate the thermal resistance of clothing. The average thermal resistance of male clothing in summer was 0.58 clo, and the average thermal resistance of female clothing was 0.6 clo. The average thermal resistance of male clothing in winter was 1 clo, and the average thermal resistance of female clothing was 1.4 clo.

#### 3.1.2 Measured climate data

As shown in Table 3, in summer, T_a_ measured outdoors was between 15.7°C and 34.6°C, V_a_ was between 0.1 m/s and 2.3 m/s, RH was between 21.4 and 79%, SR was between 46 w/m^2^ and 1,500w/m^2^, T_mrt_ was between 17.3°C and 44.8°C, and PET was between 9.7°C and 37.2°C. In winter, T_a_ measured outdoors was between −0.7°C and 24.6°C, V_a_ was between 0.1 m/s and 2.7 m/s, RH was between 2 and 36%, SR was between 15 w/m^2^ and 880 w/m^2^, T_mrt_ was between −0.5°C and 31°C, and PET was between −5.4°C and 23.7°C.

**Table 3:** Statistical value of meteorological factors measured in winter and summer.

		Ta (°C)	Va (m/s)	RH (%)	SR (w/m^2^)	Tmrt (°C)	PET (°C)
Summer	Min.	15.7	0.1	21.4	46	17.3	9.7
	Mean	25.2	0.9	45.6	389.6	28.2	24.7
	Max.	34.6	2.3	79	1500.0	44.8	37.2
	SD	3.4	0.5	13.4	328.5	6.1	27.2
Winter	Min.	−0.7	0.1	2	15	−0.5	−5.4
	Mean	11.9	0.9	13.9	527.6	17.9	10.8
	Max.	24.6	2.7	36	880.0	31	23.7
	SD	4.6	0.5	8.4	254.9	6.3	6.9

#### 3.1.3 Subjective thermal sensation

**Figure 3:**
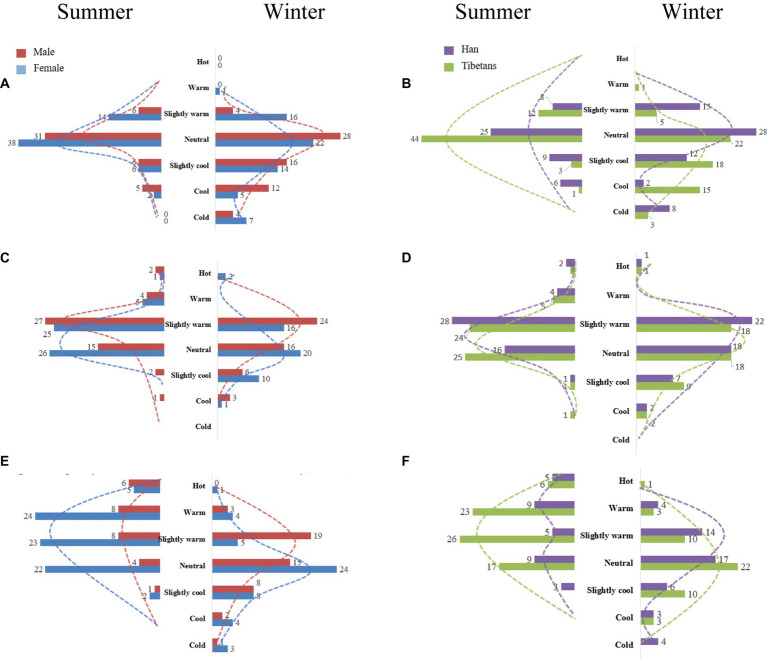
Thermal sensation responses of students of different genders and nationalities under different activity intensities. **(A)** Frequency of thermal sensation at light intensity (gender). **(B)** Frequency of thermal sensation at light intensity (nationality). **(C)** Frequency of thermal sensation at moderate intensity (gender). **(D)** Frequency of thermal sensation at moderate intensity (nationality). **(E)** Frequency of thermal sensation at vigorous intensity (gender). **(F)** Frequency of thermal sensation at vigorous intensity (nationality).

Under the light-intensity activity, it can be seen from Figure 3A that in summer, most men indicating being neutral, slightly warm, or hot, and most women indicated being slightly warm or warm. However, in winter, most men indicated being neutral, while most women indicated being slightly warm. From Figure 3B, it can be seen that in summer, most Tibetan college students indicated that they were slightly warm and warm, and in winter, they mostly indicated that they were neutral. Han college students’ thermal sensation was slightly warm or neutral in summer and neutral or slightly warm in winter. Therefore, under light-intensity activity, women may feel hotter than men in winter and summer. Moreover, Tibetan students were more likely to feel hot than Han students in summer, and Han students were more likely to feel hot than Tibetan students in winter.

It can be seen from Figure 3C that in summer, under moderate-intensity activity, most men indicated that they were neutral or slightly warm, and most women indicated that they were slightly warm. In winter, the thermal sensation of men was mainly neutral, while that of women was mainly slightly warm. From Figure 3D, it can be seen that Tibetan and Han college students’ thermal sensations were mainly neutral or slightly warm in summer. In winter, students felt mainly neutral or slightly warm. Therefore, under moderate-intensity activity, women may be more likely to feel hot than men in winter and summer, and there appeared to be little difference in thermal sensation between Tibetan and Han college students.

Under vigorous-intensity activity, it can be seen from Figure 3E that the thermal sensations of males and females in winter and summer were mainly neutral. From Figure 3F, it can be seen that the thermal sensations of Tibetan and Han college students in winter and summer were mainly neutral. Therefore, under vigorous-intensity activities, there may be little difference in gender and thermal sensation between winter and summer.

In summary, it may be because Tibetans in Tibet have lived in extreme climates at high altitudes for long time. Tibetan people are more sensitive to environmental changes, and Han people are more adapted to the temperate climate of the Central Plains, and their perception of cold and heat is not obvious. In addition, physiological differences between men and women may also lead to differences in thermal sensation.

### 3.2 Correlation between PET and thermal sensation under different personal factors

To further determine the influence of gender and nationality on thermal sensation, the regression analysis of PET with 3°C effective temperature intervals and corresponding average thermal sensation was carried out using the bin method, which is a discretization method for converting decimal numbers into binary numbers. Because grouping by gender and ethnic group will reduce the amount of data in each group, every 3°C as an interval can increase the amount of data in each group.

#### 3.2.1 Gender

**Figure 4:**
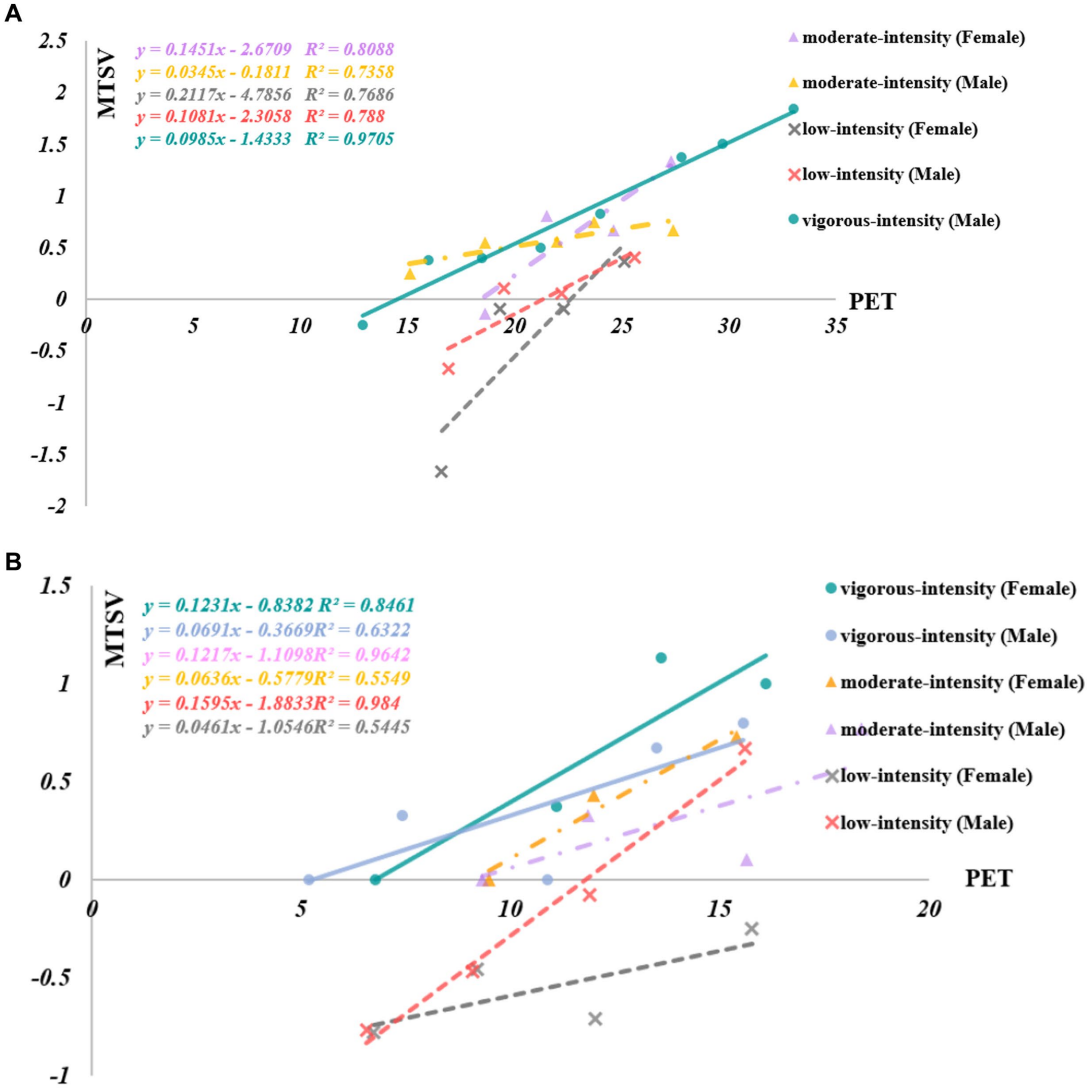
**(A)** Regression relationship between PET and MTSV in terms of the gender of students under different activity intensities during summer. **(B)** Regression relationship between PET and MTSV in terms of the gender of students under different activity intensities during winter.

As shown in Figures 4A,B, under high-intensity activities, men were more sensitive to the outdoor thermal environment than women in summer, while in winter, women felt warmer than men when the PET temperature was above 8.7°C.

Under moderate-intensity activities, women were more sensitive to the thermal environment than men in winter and summer. Under light-intensity activities, women were more sensitive to the outdoor thermal environment than men in summer, while in winter, the opposite was true.

#### 3.2.2 Nationality

**Figure 5:**
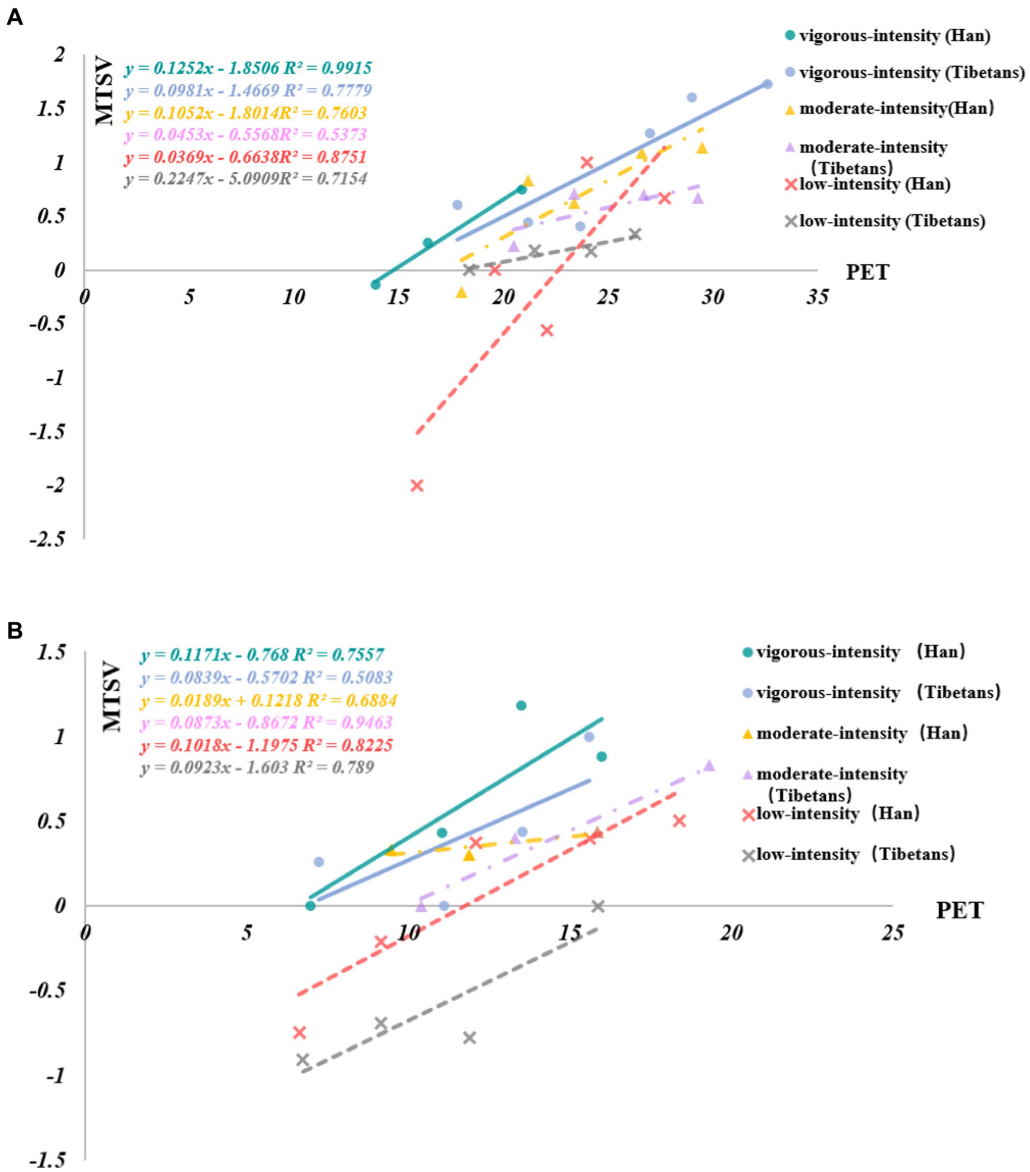
**(A)** Regression relationship between PET and MTSV in terms of nationality under different activity intensities during summer. **(B)** Regression relationship between PET and MTSV in terms of nationality under different activity intensities during winter.

As shown in Figures 5A,B, under high-intensity activities, Han Chinese college students were more sensitive to the outdoor thermal environment than Tibetan college students in both winter and summer. However, these results are inconsistent with the conclusion in Section 3.1.3. Under high-intensity activities, Tibetan college students may be more likely to feel hot than Han college students in summer, while the opposite was true in winter.

Under moderate-intensity activities, Han Chinese college students were more sensitive to the outdoor thermal environment than Tibetan college students in summer, while the opposite was true in winter, contradicting the conclusion in Section 3.1.3. Under moderate-intensity activities, there was little difference in thermal sensation between Tibetan and Han university students in winter and summer.

Under light-intensity activities, the sensitivity of Han Chinese college students to the outdoor thermal environment was higher than that of Tibetan college students in summer, while there was no significant difference in winter. This result is inconsistent with the statistical analysis results given in Section 3.1.3. Under light-intensity activities, there was no significant difference in thermal sensation between Tibetan and Han university students in winter and summer.

### 3.3 Correlation between the subjective thermal response and different climatic factors

In this section, we study the influence of various factors and the variability of the microclimate on the subjective thermal response under different intensity activities through Spearman correlation analysis.

As shown in Table 4, overall, the correlation of T_a_ and T_s_ with thermal sensation was more significant than that of V_a_ and RH with thermal sensation in winter and summer. Under light-intensity activities, T_a_ and T_s_ were significantly correlated with thermal sensation in winter and summer. Under moderate-intensity activities, T_a_ and RH were not correlated with thermal sensation in winter. Under high-intensity activities, only T_a_ was not correlated with thermal sensation in summer, and only RH was not correlated with thermal sensation in winter.

**Table 4:** Correlation analysis between microclimate elements and thermal sensation.

		T_a_ (°C)	T_s_ (°C)	V_a_ (m/s)	RH (%)
Summer	Vigorous	0.542^**^	0.760^**^	−0.077	−0.291^**^
	Moderate	0.312^**^	0.324^**^	−0.211^*^	−0.196^*^
	Light	0.295^**^	0.471^**^	0.158	−0.178
Winter	Vigorous	0.264^*^	0.306^**^	−0.316^**^	−0.041
	Moderate	0.257^*^	0.383^**^	−0.042	−0.010
	Light	0.294^**^	0.443^**^	0.027	−0.040

**Significant correlation at 0.01 (two-tailed)/ *Significant correlation at 0.05 (two-tailed).

Under vigorous-intensity activities, PET had the greatest impact on thermal sensation in winter and summer. Under moderate-intensity activity, PET had the greatest influence on thermal sensation in summer, and T_mrt_ had the greatest influence on thermal sensation in winter. Under light-intensity activities, T_mrt_ had the greatest impact on thermal sensation in winter and summer. It is worth noting that T_a_, T_g_, SR, T_mrt_, and PET had a strong influence on thermal sensation under vigorous-intensity activities in summer.

### 3.4 Thermal benchmark under different intensity activity levels

To obtain the neutral PET, comfortable PET, and the most acceptable PET of the Tibetan college students in winter and summer, the thermal sensation evaluation model, thermal comfort evaluation model, and thermal acceptability evaluation model of the Tibetan college students were established by linear regression. In the regression equation, the mean thermal sensation vote (MTSV), mean thermal comfort vote (MTCV), and mean thermal acceptable vote (MTAV) were used as the dependent variables of the subjects. The bin method was used to take PET at 1°C effective temperature intervals to offset the individual differences of the subjects. The average PET in each effective temperature interval was used as an independent variable to regress against the dependent variable.

#### 3.4.1 Neutral PET

The description of the fitting equation and regression analysis of neutral PET are as follows. From the fitting equation, it can be seen that the R2 of the equation was higher than the ordinary level, indicating that the equation had a better fit to the data. When the thermal sensation of the subject was 0, the corresponding temperature was defined as a neutral PET. According to the calculation, the neutral PET of Tibetan college students during outdoor activities in summer was 16.3°C, and the neutral PET of outdoor activities in winter was 12.1°C.

**Figure 6:**
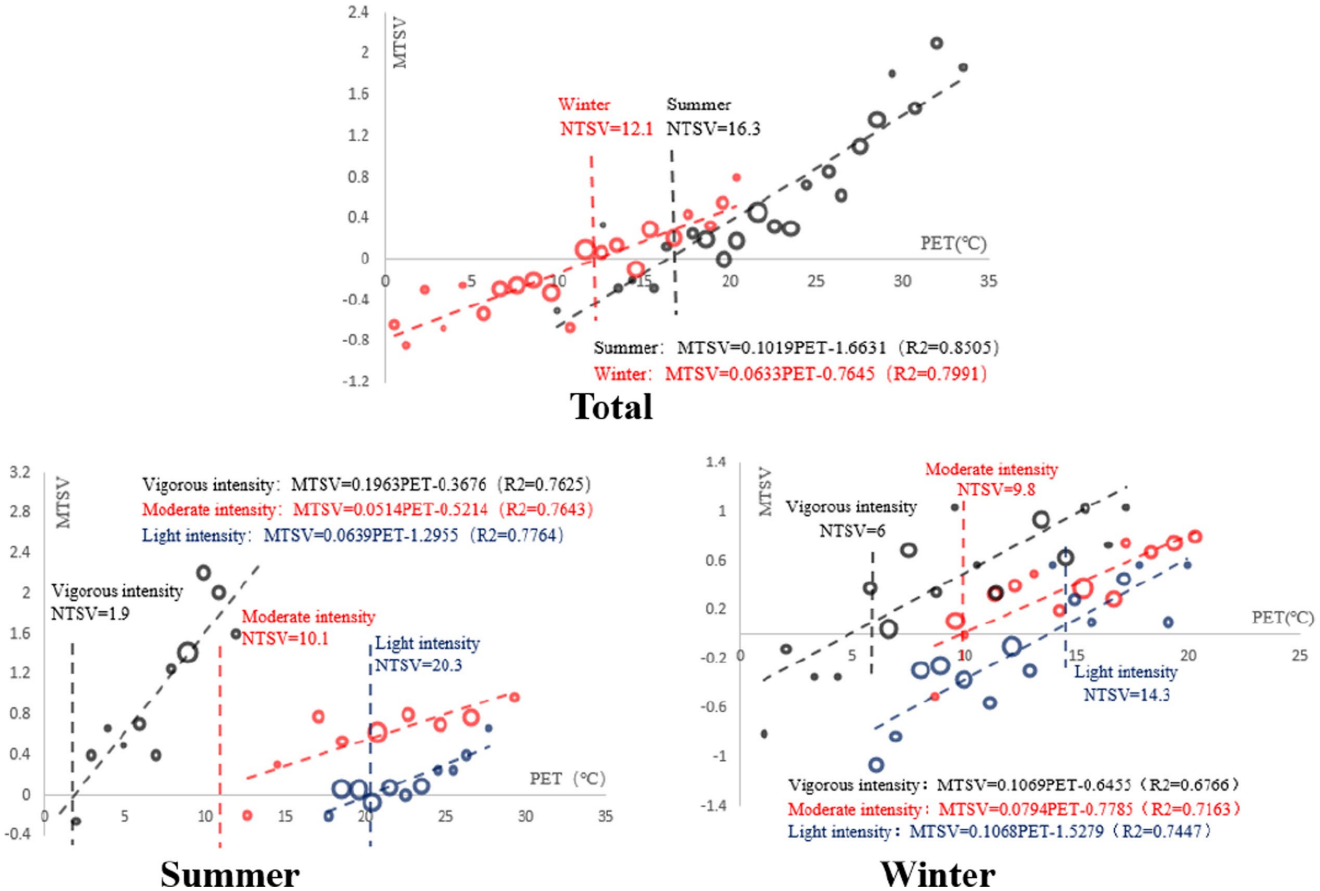
Regression analysis of MTSV and PET in winter and summer.

In summer, the neutral PET of vigorous-intensity activity was 1.9°C, the neutral PET of moderate-intensity activity was 10.1°C, and the neutral PET of light-intensity activity was 20.3°C. In winter, the neutral PET of vigorous-intensity activity was 6.0°C, the neutral PET of moderate-intensity activity was 9.8°C, and the neutral PET of light-intensity activity was 14.3°C. It can be seen from the regression that the neutral PET decreased with the increase in activity intensity (Figure 6).

#### 3.4.2 Comfortable PET

**Figure 7:**
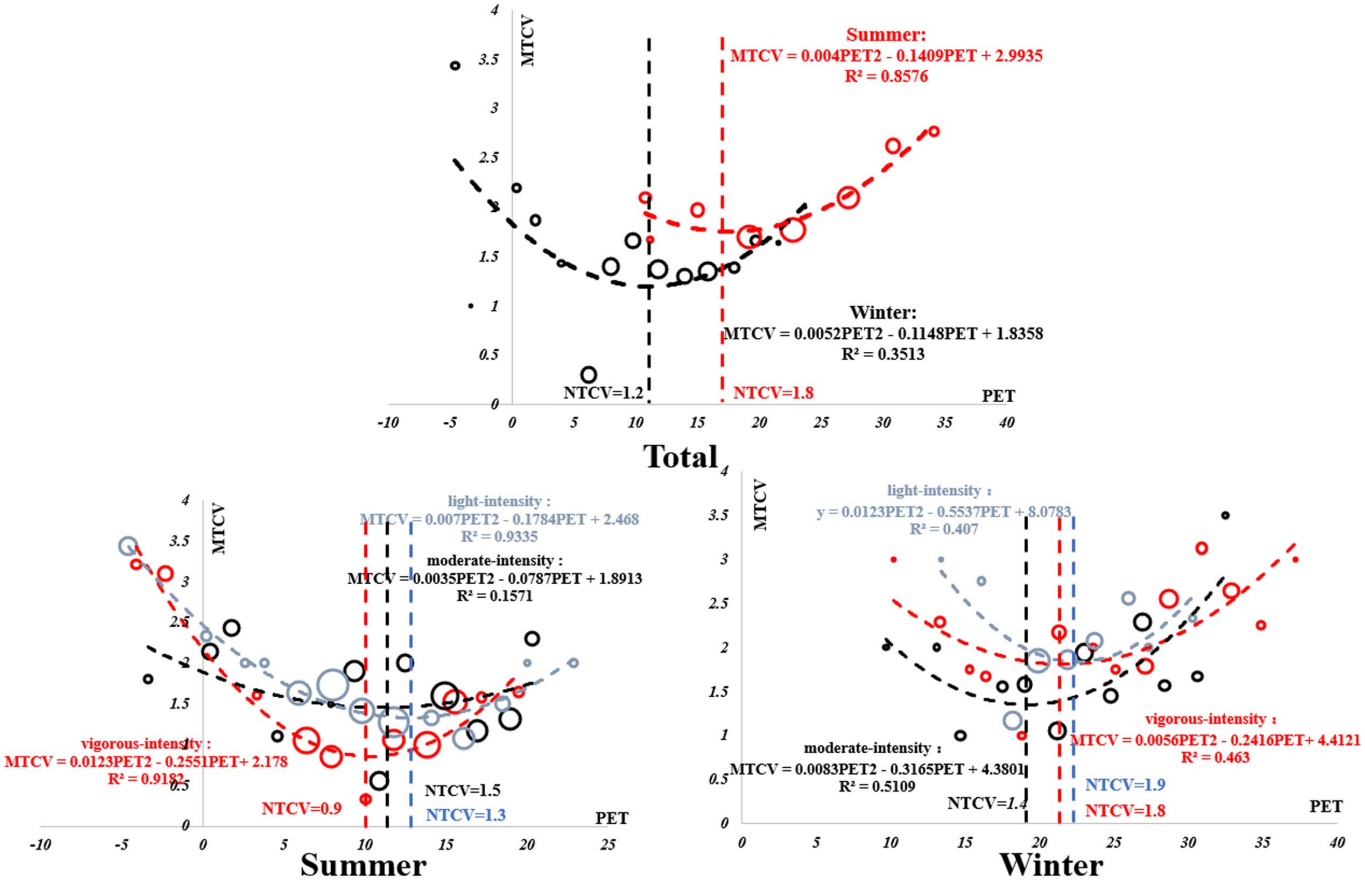
Regression analysis of MTCV and PET in winter and summer.

The fitting equation and regression analysis of comfortable PET are as follows. Unlike the relationship between MTSV and PET, MTCV and PET had a quadratic function relationship. According to the questionnaire setting and the nature of the quadratic function, when the equation reached the minimum value, the corresponding PET was the most comfortable PET. According to the calculation, the most comfortable PET value of Tibetan college students for outdoor activities in summer was 17.6°C, and the most comfortable PET for outdoor activities in winter was 11.5°C. For vigorous-intensity activity, the comfort PET was 21.6°C in summer and 10.4°C in winter. In the case of moderate-intensity activity, the comfort PET was 19.0°C in summer and 11.0°C in winter. For light-intensity activity, the comfort PET was 22.5°C in summer and 12.7°C in winter. It can be seen from the fitted regression diagram that the comfort PET gradually increased with the increase in activity intensity in summer, while the comfort PET gradually decreased with the increase in activity intensity in winter (Figure 7).

#### 3.4.3 TAV and PET

**Figure 8:**
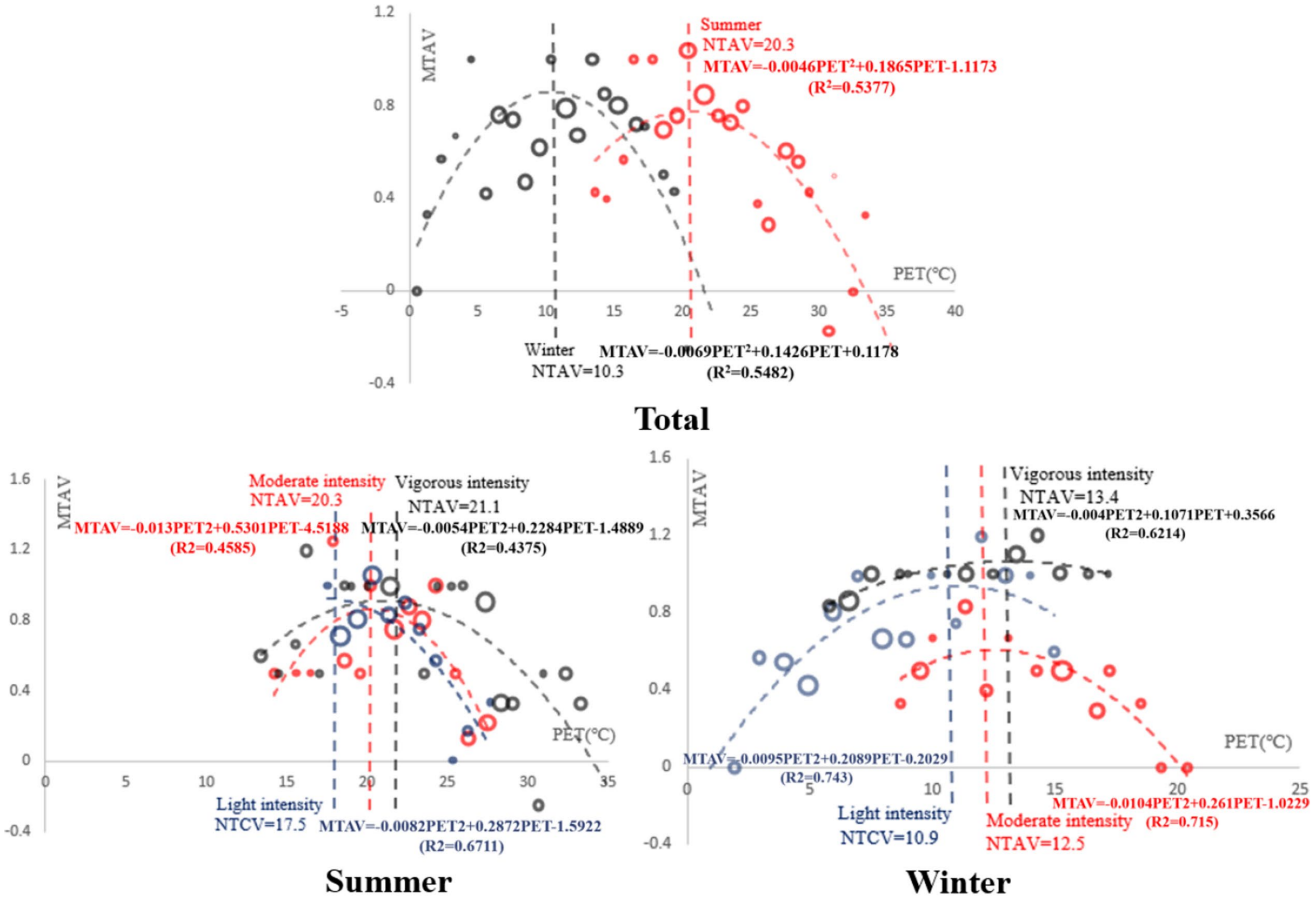
Regression analysis of MTAV and PET in winter and summer.

The fitting equation and regression analysis of the most acceptable PET are as follows. MTAV and PET had a quadratic function relationship. Using the same calculation method, the highest acceptance PET value of outdoor activities for Tibetan college students in summer was 20.3°C, and the highest acceptance PET value of outdoor activities in winter was 10.3°C. For vigorous-intensity activity, the highest acceptance PET value in summer was 21.1°C, and the highest acceptance PET value in winter was 13.4°C. For moderate-intensity activity, the highest acceptance PET value in summer was 20.3°C, and the highest acceptance PET value in winter was 12.5°C. During light-intensity activities, the highest acceptance PET value in summer was 17.5°C, and the highest acceptance PET value in winter was 10.9°C (Figure 8).

### 3.5 TCV and TSV

**Figure 9:**
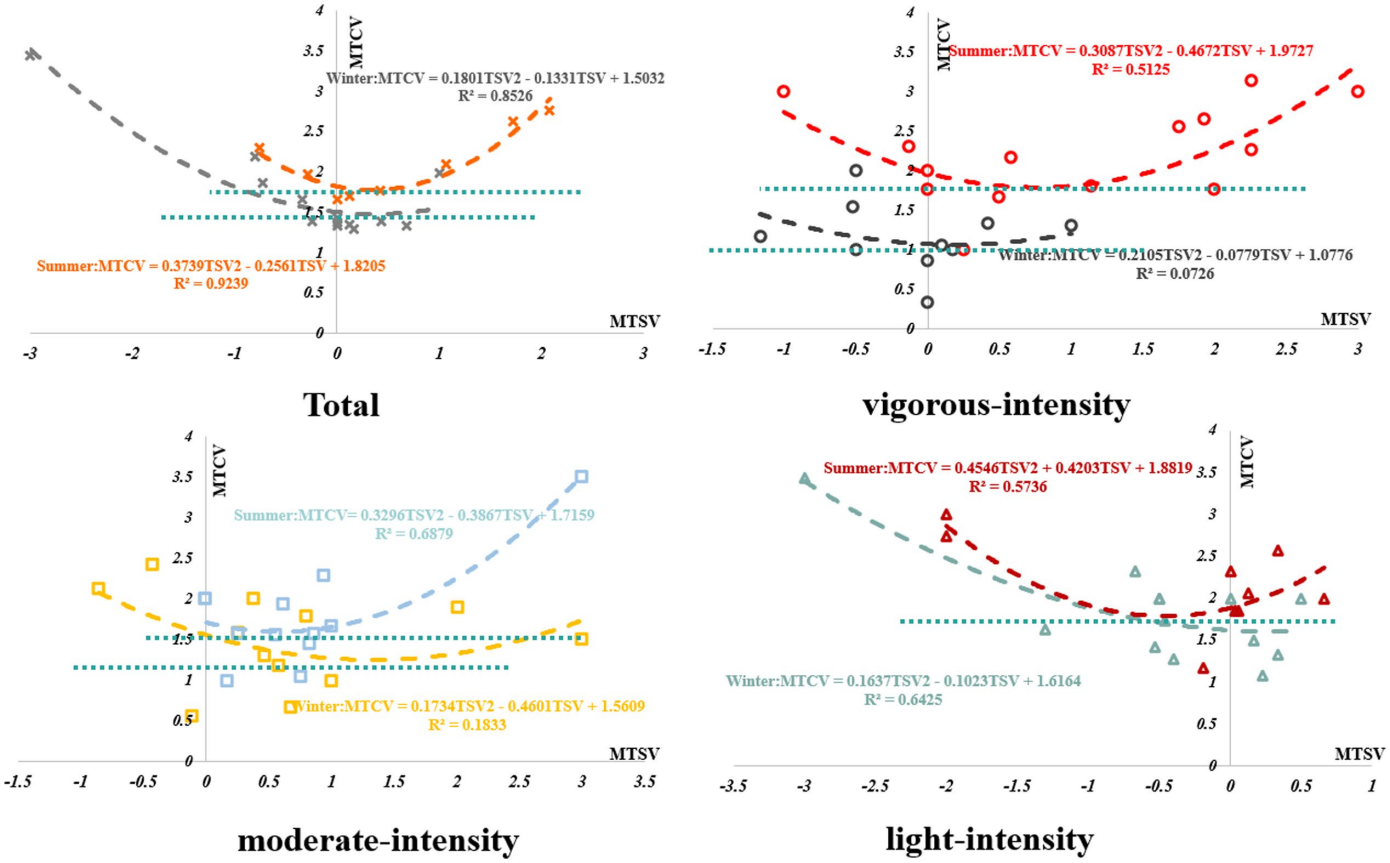
Regression analysis of TSV and TCV in winter and summer.

From Sections 3.4.1 and 3.4.2, it can be inferred that neutral PET differed significantly from comfortable PET. To obtain the relationship between the thermal sensation and thermal comfort of college students under different intensity activities in winter and summer, regression analysis was conducted on the thermal sensation TSV and average thermal comfort MTCV of Tibetan college students in winter and summer to compare and explore the specific relationship between them, as shown in Figure 9.

According to Figure 9, except for light-intensity activities, thermal sensation TSV and average thermal comfort MTCV had a quadratic function relationship. In summer, the most comfortable state MTCV = 1.8 was reached when the TSV was 0.4; in winter, the most comfortable state MTCV = 1.3 was reached when the TSV was 0.5. In winter, the thermal sensation reached the most comfortable state between neutral and slightly warm, and in summer, the thermal sensation reached the most comfortable state between slightly cold and slightly warm. Under vigorous-intensity activity, in summer, when TSV = 0.8 and MTCV = 1.8, the thermal sensation reached the most comfortable state; in winter, when TSV = 0.3 and MTCV = 1, the thermal sensation reached the most comfortable state. Under moderate-intensity activity, in summer, when TSV = 0.7 and MTCV = 1.7, students’ thermal sensation reached the most comfortable state; in winter, when TSV = 1.5 and MTCV = 1.3, their thermal sensation reached the most comfortable state. Under light-intensity activity, in summer, when TSV = −0.5 and MTCV = 1.8, students’ thermal sensation reached the most comfortable state.

Therefore, the relationship between thermal comfort and thermal sensation was different under different activity intensities. Under vigorous-intensity activities, in winter and summer, the thermal sensation of students reached the most comfortable state between neutral and slightly warm. Under moderate-intensity activities, in winter and summer, the thermal sensation reached the most comfortable state between neutral and slightly warm. Under light-intensity activities, the most comfortable state was not found in winter; the higher the thermal sensation, the more comfortable it was. Furthermore, the summer thermal sensation reached the most comfortable state between neutral and slightly cold.

## 4 Discussion

### 4.1 Comparison with previous studies

#### 4.1.1 Thermal comfort and neutral PET and its range

The thermal sensation and thermal comfort of college students under different activity intensities show a quadratic function relationship. When the most comfortable state is reached in winter, the TSV values are 1.5 (medium intensity) and 0.3 (heavy intensity), respectively. When the summer reaches the most comfortable state, the TSV values are −0.5 (light intensity), 0.7 (medium intensity), and 0.4 (heavy intensity), respectively. It can be seen that the TSV changes greatly in winter and little in summer, which is consistent with the study of Hot summer and cold winter zone (20). The higher the temperature, the smaller the effect of activity intensity on thermal comfort.

In the regression equation of neutral PET, with the intensity of activity from mild to moderate to severe, the neutral PET values in summer were 20.3 ° C, 10.1 ° C, 1.9 ° C, and the neutral PET values in winter were 14.3 ° C, 9.8 ° C, 6.0 ° C. It can be seen that the neutral PET value gradually decreases with the increase of activity intensity. Niu et al., in the study of campus thermal comfort in Xi ‘an (28), with the activity intensity from mild to moderate to severe, the summer neutral PET values were 26.1 ° C, 22.1 ° C, 11.9 ° C (28). It can be seen that the neutral PET values gradually decrease with the increase of activity intensity, which is verified in both studies. Although both studies were carried out in the cold climate zone, the neutral PET values under different activity intensities also differed by nearly 10 ° C. This is because Tibet is located in a high altitude area, and local college students have a stronger ability to withstand cold.

There are great differences in outdoor thermal neutral temperature in different climatic regions. This paper takes light intensity as an example (Table 5). When college students in high-altitude and cold areas carried out light-intensity activities, the neutral PET in summer was 20.3°C, the comfortable PET was 20.0°C, and the PET with the highest acceptance was 17.5°C. In winter, the neutral PET was 14.3°C, the comfortable PET was 14.7°C, and the PET with the highest acceptance was 10.9°C. In hot summer and cold winter regions of China, the neutral PET in winter and summer are 23.5°C and 22.8°C, respectively, and the average solar radiation level in summer (110 W/m^2^) is higher than that in winter (96 W/m^2^). The most comfortable PET in winter is 30.4°C, and the most comfortable PET was not found in summer (7). A study conducted at a university in Harbin, a cold climate zone in China, showed that the neutral PETs in summer and winter were 20.0°C and 18.0°C, respectively (35). In an outdoor study conducted in a humid subtropical campus, it was found that the neutral PET of light-intensity activity, moderate-intensity activity, and vigorous-intensity activity in summer was 11.9°C, 22.1°C, and 26.1°C, respectively, while the neutral PET of light-intensity activity, moderate-intensity activity, and vigorous-intensity activity in summer was 1.9°C, 10.1°C, and 20.3°C, respectively (28).

**Table 5:** Comparison of research conclusions.

	Climate type	PET	Activity intensity	Gender	Nationality
This study	High-altitude cold zone	Neutral PET: 14.3°C, 20.3°C; comfort PET: 13.6°C, 21.5°C	Relational	Under heavy- and medium-intensity activities, women are more sensitive than men Under light-intensity activities, women are more sensitive to the outdoor thermal environment than men in summer, and men are more sensitive to the outdoor thermal environment than women in winter	Under heavy- and light-intensity activities, the sensitivity of Han nationality students is higher than that of Tibetan nationality students Under moderate-intensity activities, the sensitivity of Han nationality students is high in summer, and that of Tibetan nationality students is high in winter
Zheng et al. (20)	Hot summer and cold winter zone	Neutral PET: 23.5°C, 22.8°C	The higher the ambient temperature, the less the influence of the activity level on the human thermal sensation	Gender may not be related to TSV and TCV	
Yang et al. (2018)	Cold zone	Neutral PET: 22.1°C	Relational		
Chen et al. (34)	Severe cold zone	Neutral PET: 18°C, 20°C	The higher the outdoor activity level, the higher the comfort level At the same time, the comfortable PET is also related to the activity time	The comfort range of men is greater than that of women, and women are highly sensitive	In winter, the outdoor activity comfort level of residents in cold areas is slightly higher than that of residents in warm areas.

#### 4.1.2 Gender and ethnicity

In the return equation between TSV and PET, with the increase of activity intensity in summer, the female’s rate is greater than that of male, and the male’s rate (1.883) is greater than that of female in light intensity activity in winter, In winter moderate intensity (0.578) and heavy intensity (0.838) activities, the slope of female is larger than that of male. It can be seen that male is more sensitive to temperature in winter light intensity activities, and female is more sensitive in other times, which is different from other Severe cold zone studies (34, 36–38). In the research of An et al., it is discussed that there are great differences in clothing thermal resistance between men and women, which has a certain impact on thermal comfort, However, there was little difference in the clothing thermal resistance between men and women in Lhasa, this may be the reason for the different conclusions from the other studies.

In the return equation between TSV and PET, under high-intensity activities, Han Chinese college students were more sensitive to the outdoor thermal environment than Tibetan college students in both winter and summer. Under moderate-intensity activities, Han Chinese college students were more sensitive to the outdoor thermal environment than Tibetan college students in summer, while the opposite was true in winter. Under light-intensity activities, the sensitivity of Han Chinese college students to the outdoor thermal environment was higher than that of Tibetan college students in summer, while there was no significant difference in winter. At present, other studies have not clearly drawn this conclusion. The above differences show that, due to the cold and cold storage areas at high altitudes for a long time, the Tibetan students in Lhasa have formed a strong cold resistance.

We have increased the research on thermal comfort in alpine areas, and solved the shortcomings of the research on the thermal comfort of college students in Tibetan areas, and improved the research on the influence of different activity intensity, gender, nationality and other factors on thermal comfort. In the future, it is necessary to increase the relevant research on the influence of different genders on thermal comfort in the plateau area, which will provide greater help for the comfort design of public space for different genders in the future.

The important factors considered in this study were activity intensity, gender, and nationality, and the results showed that there were differences in thermal sensation among different genders and ethnic groups under different activity intensities. Because most of the previous OTC studies were conducted under light-intensity activities (i.e., sitting or standing), herein we studied the thermal comfort of college students during light-intensity exercises.

### 4.2 Design strategies

**Figure 10:**
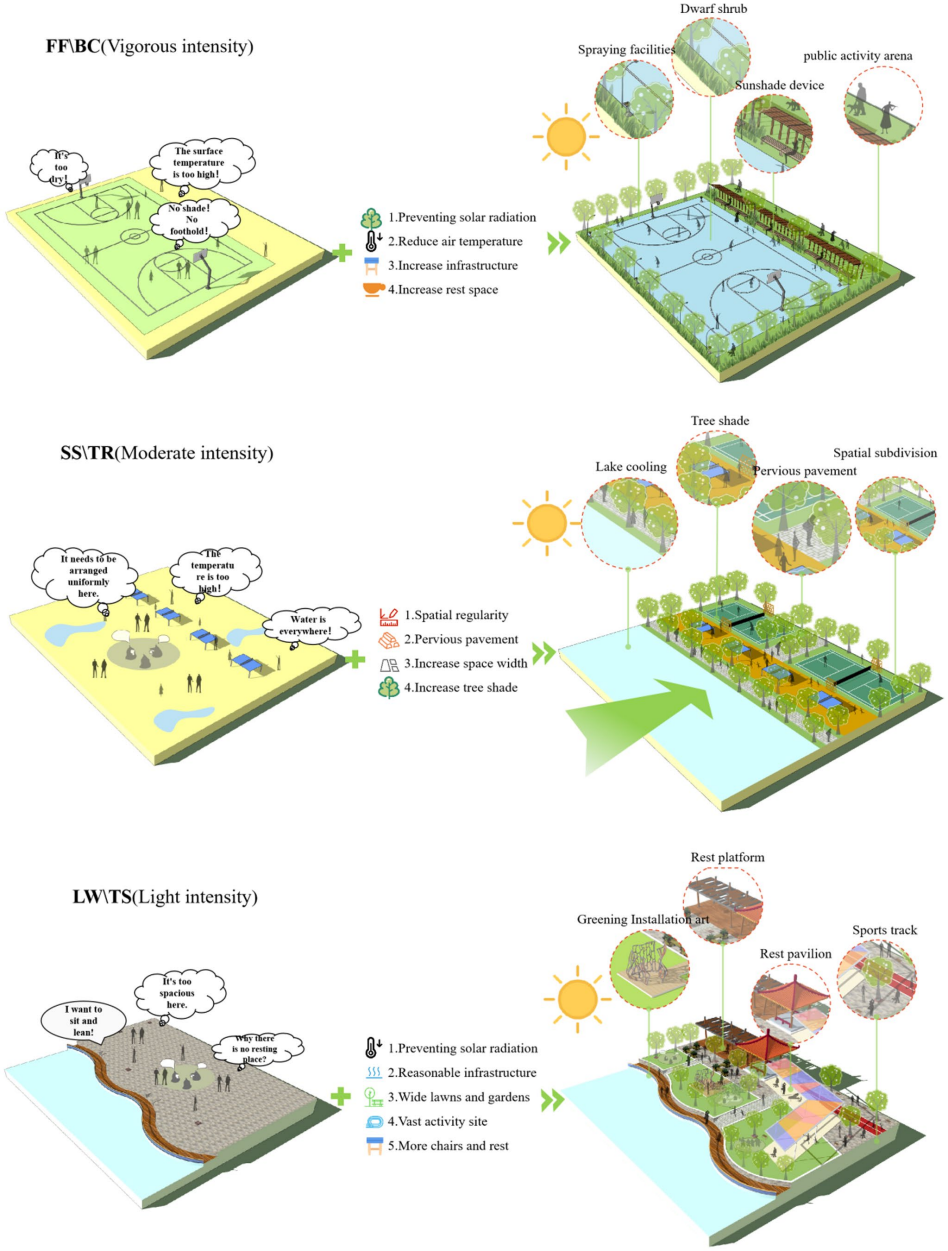
Analysis of design strategies for different activity intensities.

The influence of climatic factors on thermal sensation under different intensity activities has been considered for the optimization of the outdoor thermal environment under different intensity activities. As shown in Figure 10, taking the FF\BC outdoor activity space as an example, for the small-scale space design under vigorous-intensity activities, tall native trees can be planted, and flowering shrubs can be configured at the lower level. On the one hand, a certain greening level can be formed (39); on the other hand, a protective belt can be formed to ensure the safety of pedestrians, and it can also reduce the impact of high-intensity activities on other outdoor spaces. The altitude of Tibet is so high, and the green plants are mostly cushion plants. Therefore, tall trees can be replaced by wooden grilles or other structures. In addition, the annual solar radiation in Tibet is very strong. Where circumstances permit, appropriate shading and spraying facilities can be added to reduce T_a_ and SR. Taking the SS\TR outdoor activity space as an example, for moderate-intensity activity space, the space can be divided into ground divisions (28), plant divisions, and various facilities to increase the space utilization and comfort of college students staying in this location. In summer, the comfort PET value of college students gradually decreases, and it is necessary to pay attention to ventilation, while in winter, the comfort PET value increases, and it is necessary to pay more attention to warmth. Furthermore, the selection of good paving materials and the planting of appropriate evergreen shade trees can reduce the temperature, wind speed, and solar radiation. Additionally, in the design process, it is necessary to pay attention to the influence of the openness of space and winter humidity. Taking the LW\TS activity space as an example, for the light-intensity activity space, it is usually focused on transparency and openness to facilitate the evacuation of people. The comfort PET value of residents in summer is lower than winter. It is necessary to increase the enclosure and tightness of the space. However, the low-intensity space is mostly evacuation space. At the same time, trees set up for shading should be able to adapt to a high-altitude climate. In addition, flower beds and enclosed seats can be set up in appropriate locations to provide college students with a place to rest (40, 41).

In a word, there are differences in thermal sensation between different genders, different nationalities and different seasons under different activity intensities. In winter, with the increase of activity intensity, it is necessary to increase the warmth of the activity space, while in summer, more attention should be paid to the ventilation of the space. Therefore, the same space type can set up multi-season activity venues to directly or indirectly adjust the space temperature and create the most suitable activity space.

## 5 Conclusion

To understand the OTC characteristics of college students from different activity intensities, so as to provide data support for the optimization strategy of OTC of college students, an experiment was conducted in the outdoor space of a university campus in a high-altitude and cold region of China. The main conclusions are as follows:

1. The neutral PET value decreased and the PET value with the highest acceptance increased with the increase in activity intensity when college students in high-altitude cold regions performed different intensity activities. In summer, as the activity intensity increased, the comfort PET gradually increased, while in winter, the PET gradually decreased. The greater the activity intensity, the greater the difference between neutral PET and comfort PET, and the greater the difference between neutral PET and the most acceptable PET.
2. There were differences between thermal comfort and thermal sensation under different activity intensities. Under vigorous and moderate intensity activities, the thermal sensation in winter and summer reached the most comfortable state between neither cold nor hot and slightly warm. Under light-intensity activities, the most comfortable state was reached between hot and very hot in winter, and the most comfortable state was reached between neither cold nor hot and slightly cold in summer.
3. In winter and summer, under vigorous-intensity activities, PET had the greatest impact on thermal sensation; under light-intensity activity, T_mrt_ had the greatest influence on thermal sensation. The situation was different under moderate-intensity activity. PET had the greatest influence on thermal sensation in summer, and T_mrt_ had the greatest influence on thermal sensation in winter.
4. The effect of gender on thermal sensation was different under different intensities. Under vigorous-intensity activities, women were more sensitive to the outdoor thermal environment than men in winter. Under moderate-intensity activity, women were more sensitive to the outdoor thermal environment than men in winter and summer. Under light-intensity activities, women were more sensitive to the outdoor thermal environment than men in summer, and men were more sensitive in winter.
5. The influence of ethnic groups on thermal sensation was different under different intensities. Under vigorous-intensity activities, Han college students were more sensitive to the outdoor thermal environment than Tibetan college students in winter and summer. Under moderate-intensity activity, Han college students had higher sensitivity in summer and Tibetan college students had higher sensitivity in winter. Under light-intensity activities, Han college students were more sensitive to the outdoor thermal environment in summer, and there was little difference in winter.

This study has some shortcomings. In the experiments conducted in this study, the Tibetan college students were not dressed uniformly, which may have introduced some errors in the experimental results. Follow-up research needs to be further improved and carried out.

## Data availability statement

The original contributions presented in the study are included in the article/supplementary material, further inquiries can be directed to the corresponding author.

## Ethics statement

The studies involving humans were approved by the ethics committee at Southwest Jiaotong University, School of Architecture. The studies were conducted in accordance with the local legislation and institutional requirements. The participants provided their written informed consent to participate in this study. Written informed consent was obtained from the individual(s) for the publication of any identifiable images or data included in this article.

## Author contributions

YZ: Writing – review & editing, Conceptualization, Data curation, Formal analysis, Funding acquisition, Methodology, Project administration, Resources, Supervision. XZ: Visualization, Formal analysis, Investigation, Software, Validation, Writing – original draft. JH: Conceptualization, Data curation, Formal analysis, Investigation, Methodology, Project administration, Software, Validation, Writing – original draft. XL: Formal analysis, Writing – review & editing.

## References

[1] Jia Y Zhou Z Cao X Hu W Xiang F Xiong L. Meta-analysis of the detection rate of sub-health status in Chinese college students _ Jia Yu. Chin J Evid Based Med. (2023) 23:901–7.

[2] Boss Data Research Center. There are 70% of sub-healthy people in China with significant differences in each category [EB/OL]. (2014). Available at: http://www.bosidata.com/baojianshichang1404/C44775DR8R.html.

[3] Jaacks LM Gordon-Larsen P Mayer-Davis EJ Adair LS Popkin B. Age, period and cohort effects on adult body mass index and overweight from 1991 to 2009 in China: the China health and nutrition survey. Int J Epidemiol. (2013) 42:828–37. doi: 10.1093/ije/dyt052, PMID: 23771721 PMC3733700

[4] Ng SW Norton EC Popkin BM. Why have physical activity levels declined among Chinese adults? Findings from the 1991-2006 China health and nutrition surveys. Soc Sci Med. (2009) 68:1305–14. doi: 10.1016/j.socscimed.2009.01.035, PMID: 19232811 PMC2731106

[5] Atkin AJ Sharp SJ Harrison F Brage S Van Sluijs EMF. Seasonal variation in Children’s physical activity and sedentary time. Med Sci Sports Exerc. (2016) 48:449–56. doi: 10.1249/MSS.0000000000000786, PMID: 26429733 PMC4762193

[6] Amindeldar S Heidari S Khalili M. The effect of personal and microclimatic variables on outdoor thermal comfort: a field study in Tehran in cold season. Sustain Cities Soc. (2017) 32:153–9. doi: 10.1016/j.scs.2017.03.024

[7] Nikolopoulou M Lykoudis S. Use of outdoor spaces and microclimate in a Mediterranean urban area. Build Environ. (2007) 42:3691–707. doi: 10.1016/j.buildenv.2006.09.008

[8] Elnabawi MH Hamza N Dudek S. Thermal perception of outdoor urban spaces in the hot arid region of Cairo, Egypt. Sustain Cities Soc. (2016) 22:136–45. doi: 10.1016/j.scs.2016.02.005

[9] Cheung PK Jim CY. Subjective outdoor thermal comfort and urban green space usage in humid-subtropical Hong Kong. Energ Buildings. (2018) 173:150–62. doi: 10.1016/j.enbuild.2018.05.029

[10] Yang W Wong NH Jusuf SK. Thermal comfort in outdoor urban spaces in Singapore. Build Environ. (2013) 59:426–35. doi: 10.1016/j.buildenv.2012.09.008

[11] Thorsson S Lindqvis M Lindqvis S. Thermal bioclimatic conditions and patterns of behavior in an urban park in Goteborg, Sweden. Int J Biometeorol. (2004) 48:149–56. doi: 10.1007/s00484-003-0189-8, PMID: 12955614

[12] Zacharias J Stathopoulos T Wu H. Microclimate and downtown open space activity. Environ Behav. (2001) 33:296–315. doi: 10.1177/0013916501332008

[13] Liu J Yang X Jiang Q Qiu J Liu Y. Occupants' thermal comfort and perceived air quality in natural ventilated classrooms during cold days. Build Environ. (2019) 158:73–82. doi: 10.1016/j.buildenv.2019.05.011

[14] Huang T Li J Xie Y Niu J Mak CM. Simultaneous environmental parameter monitoring and human subject survey regarding outdoor thermal comfort and its modelling. Build Environ. (2017) 125:502–14. doi: 10.1016/j.buildenv.2017.09.015

[15] Zhao L Zhou X Li L He S Chen R. Study on outdoor thermal comfort on a campus in a subtropical urban area in summer. Sustain Cities Soc. (2016) 22:164–70. doi: 10.1016/j.scs.2016.02.009

[16] Xi T Li Q Mochida A Meng Q. Study on the outdoor thermal environment and thermal comfort around campus clusters in subtropical urban areas. Build Environ. (2012) 52:162–70. doi: 10.1016/j.buildenv.2011.11.006

[17] Hallgren M Stubbs B Vancampfort D Lundin A Jääkallio P Forsell Y. Treatment guidelines for depression: greater emphasis on physical activity is needed. Eur. Psychiat. (2017) 40:1–3. doi: 10.1016/j.eurpsy.2016.08.011 27837666

[18] Hallgren M Vancampfort D Owen N Rossell S Dunstan DW Bellocco R. Prospective relationships of mentally passive sedentary behaviors with depression: mediation by sleep problems. J. Affect. Disord. (2020) 265:538–544. doi: 10.1016/j.jad.2019.11.088 31784118

[19] Friedenreich CM Pader J Barberio AM Ruan Y Poirier AE Grevers X. Estimates of the current and future burden of cancer attributable to sedentary behavior in Canada. Prev. Med. (2019) 122:73–80. doi: 10.1016/j.ypmed.2019.03.009 31078175

[20] Zheng TQ. Effect of activity level on human thermal sensation. Chongqing: Chongqing University (2014).

[21] Leng H Liang S Yuan Q. Outdoor thermal comfort and adaptive behaviors in the residential public open spaces of winter cities during the marginal season. Int J Biometeorol. (2020) 64:217–29. doi: 10.1007/s00484-019-01709-x, PMID: 30923891

[22] Shooshtarian S Ridley I. The effect of individual and social environments on the users thermal perceptions of educational urban precincts. Sustain Cities Soc. (2016) 26:119–33. doi: 10.1016/j.scs.2016.06.005

[23] Fei Y Fang H Han J Zhang Y. Study on the outdoor thermal comfort evaluation of the elderly in the Tibetan plateau, sustainable cities and society. Sustain Cities Soc. (2022) 77:103582. doi: 10.1016/j.scs.2021.103582

[24] Zhang YZ Chen LX Sun C Fu Y Xie Y. An investigation of the influence of the morphological indexes of trees on the outdoor microclimate at high altitude in summer. Front Environ Sci. (2023) 11:1098966. doi: 10.3389/fenvs.2023.1098966

[25] Ainsworth BE Haskell WL Herrmann SD Meckes N Bassett DR Jr Tudor-Locke C. 2011 compendium of physical activities: a second update of codes and MET values. Med Sci Sports Exerc. (2011) 43:1575–81. doi: 10.1249/MSS.0b013e31821ece12, PMID: 21681120

[26] Goto T Toftum J de Dear R Fanger PO. Thermal sensation and thermophysiological responses to metabolic step-changes. Int J Biometeorol. (2006) 50:323–32. doi: 10.1007/s00484-005-0016-5, PMID: 16408171

[27] Huang Z Cheng B Gou Z Zhang F. Outdoor thermal comfort and adaptive behaviors in a university campus in China's hot summer-cold winter climate region. Build Environ. (2019) 165:106414–4. doi: 10.1016/j.buildenv.2019.106414

[28] Niu J Hong B Geng Y Mi J He J. Summertime physiological and thermal responses among activity levels in campus outdoor spaces in a humid subtropical city. Sci Total Environ. (2020) 728:138757. doi: 10.1016/j.scitotenv.2020.138757, PMID: 32361116

[29] Kántor N Unger J. The most problematic variable in the course of human-biometeorological comfort assessment — the mean radiant temperature. Open Geosciences, (2011) 3:90–100. doi: 10.2478/s13533-011-0010-x

[30] Matzarakis A Rutz F Mayer H. Modelling radiation fluxes in simple and complex environments—application of the RayMan model. Int J Biometeorol (2007) 51:323–334. doi: 10.1007/s00484-006-0061-8 17093907

[31] Andréas H Alfonso V. The Kendall and spearman rank correlations of the bivariate skew normal distribution. Scand J Stat. (2022) 49:1669–98. doi: 10.1111/sjos.12587

[32] Esra E. Ulaş K a.a new Liu-type estimator in binary logistic regression models. Commun Stat. (2022) 51:4370–94. doi: 10.1080/03610926.2020.1813777

[33] Ashrae Standard 55. Thermal environmental conditions for human occupancy. Atlanta, GA: ANSI/ASHRAE Standard (2017).

[34] Chen L Zhang Y Han J Li X. An investigation of the influence of ground surface properties and shading on outdoor thermal comfort in a high-altitude residential area. Front. Archit. Res. (2021) 10:432–446. doi: 10.1016/j.foar.2020.12.005

[35] Chen X Xue P Liu L Gao L Liu J. Outdoor thermal comfort and adaptation in severe cold area: a longitudinal survey in Harbin, China. Build Environ. (2018) 143:548–60. doi: 10.1016/j.buildenv.2018.07.041

[36] An L Hong B Cui X Geng Y Ma X. Outdoor thermal comfort during winter in China's cold regions: a comparative study. Sci Total Environ. (2021) 768:144464. doi: 10.1016/j.scitotenv.2020.144464 33454480

[37] Jin H Wang B Han B. Study on environment regulation of residential in severe cold area of China in winter: Base on outdoor thermal comfort of the elderly. Sustainability. (2019) 11:6509. doi: 10.3390/su11226509

[38] Jin H Wang B Qiao L. Studies of elderly thermal comfort in outdoor environments in severe cold area of China In: Proceedings of the international conference on sustainability in energy and buildings. Cham: Springer (2018). 32–42.

[39] Zhang J Gou Z Lu Y Lin P. The impact of sky view factor on thermal environments in urban parks in a subtropical coastal city of Australia. Urban For Urban Gree. (2019) 44:126422. doi: 10.1016/j.ufug.2019.126422

[40] Watanabe S Nagano K Ishii J Horikoshi T. Evaluation of outdoor thermal comfort in sunlight, building shade, and pergola shade during summer in a humid subtropical region. Build Environ. (2014) 82:556–65. doi: 10.1016/j.buildenv.2014.10.002

[41] Ma X Tian Y Du M Hong B Lin B. How to design comfortable open spaces for the elderly? Sci Total Environ. (2021) 768:144985. doi: 10.1016/j.scitotenv.2021.144985, PMID: 33736312

